# Pharmacokinetic Parameters of Oral Firocoxib, Oral Meloxicam, and Transdermal Flunixin in Meat Type Goats

**DOI:** 10.1111/jvp.70035

**Published:** 2025-12-01

**Authors:** Mikaela M. Weeder, Michael D. Kleinhenz, Christopher T. Culbertson, Emily J. Reppert, Kushan Kompalage, Ryan Tucker, Misty Bear, Andrew K. Curtis, Ally A. Nelson, Bailey R. Fritz, Payton Dahmer, Johann F. Coetzee

**Affiliations:** ^1^ Department of Anatomy and Physiology, College of Veterinary Medicine Kansas State University Manhattan Kansas USA; ^2^ Veterinary Education, Research, and Outreach (VERO), College of Veterinary and Biomedical Sciences Texas A&M University Canyon Texas USA; ^3^ Department of Chemistry, College of Arts and Sciences Kansas State University Manhattan Kansas USA; ^4^ Department of Clinical Sciences, College of Veterinary Medicine Kansas State University Manhattan Kansas USA; ^5^ Department of Animal Sciences, College of Agriculture Kansas State University Manhattan Kansas USA

**Keywords:** firocoxib, flunixin, goats, meloxicam, pharmacokinetics

## Abstract

The objective of this study was to determine and compare the pharmacokinetics of oral firocoxib, oral meloxicam, and transdermal flunixin (TD) in 44 adult, male castrated, crossbred goats. Pharmacokinetic (PK) analysis was performed for each goat in each phase using non‐compartmental methods with descriptive statistics reported. Mean plasma half‐life (T_1/2_ (h)) for oral (PO) administration of firocoxib at varying dosages in meat type castrated goats was reported at 0.5 mg/kg, 1.0 mg/kg, and 2.0 mg/kg at 9.1 (range 6.9–13.4), 10.2 (range 6.4–14.7) and 9.2 (range 6.8–12.8), respectively. For oral meloxicam, mean plasma T_1/2_ at doses of 1.0 mg/kg, 2.0 mg/kg, and 3.0 mg/kg were 13.3 (range: 10.1–22.3), 13.1 (range: 12–24), and 11.7 (range: 8.3–20.5) hours, respectively. Transdermal flunixin showed mean plasma T_1/2_ of 16.5 (range: 10.7–62) at 3.3 mg/kg, 22.0 (range: 16.6–67.4) at 4.2 mg/kg, and 17.8 (range: 7.4–56.3) at 5.0 mg/kg. These results highlight significant variability in drug disposition and suggest that further research is warranted to optimize dosing regimens for oral firocoxib, oral meloxicam, and transdermal flunixin in goats.

## 1 Introduction

Firocoxib, meloxicam, and flunixin are non‐steroidal anti‐inflammatory drugs (NSAIDs) commonly used for their anti‐inflammatory and analgesic properties in veterinary species. Currently, there are no analgesic drugs approved by the US Food and Drug Administration (FDA) for use in caprine patients. However, veterinarians can prescribe NSAIDs to caprine patients under the Animal Medicinal Drug Use Clarification Act of 1994 with a valid veterinary‐client‐patient relationship (FDA 2024).

In the United States, firocoxib and meloxicam are approved for use in companion animals and equine patients. Both drugs are available in oral formulations that allow for ease of administration. Flunixin is formulated for both intravenous injectable and pour‐on (transdermal) use. Transdermal flunixin is approved in the United States for beef and dairy cattle to control pyrexia associated with bovine respiratory disease and mastitis, and pain control for interdigital phlegmon (foot rot) (Health 2024). These drugs were chosen based on their current availability in veterinary clinics across the United States, their ease of administration to patients, and their affordable economic cost for producers.

In goats, the pharmacokinetic data available regarding firocoxib, meloxicam, and flunixin transdermal in goats is limited to single‐dose descriptive studies (Stuart et al. 2019; Ingvast‐Larsson et al. 2011; Karademir et al. 2016; Reppert et al. 2019). Due to a lack of species‐specific data, veterinarians commonly rely on data regarding dosing, administration route, and indications extrapolated from cattle and sheep studies when treating goat patients. When treating goats, veterinarians commonly extrapolate dose, route, and induction from product labels in other livestock species. Further research into species‐specific pharmacokinetic parameters is necessary.

The objective of this study was to describe the pharmacokinetics of oral firocoxib, oral meloxicam, and flunixin transdermal at three varying dosages for each drug in castrated male meat‐type goats with experimentally induced lameness.

## 2 Materials and Methods

### 2.1 Ethics Statement

This study was approved by the Institutional Animal Care and Use Committee at Kansas State University (Protocol #4387).

### 2.2 Experimental Animals

Forty‐four castrated, Spanish‐influenced crossbred meat‐type goats between 9 and 12 months of age, with an average body weight of 35 kg (range: 25–43 kg), were enrolled in the study. Prior to enrollment, all animals were evaluated and deemed free of illness or injury by a licensed veterinarian.

### 2.3 Housing and Husbandry

Goats were individually housed (with visual access to conspecifics) in raised pens within an enclosed barn at the Kansas State Sheep and Meat Goat Unit. Animals were provided ad libitum access to water and fed brome hay twice daily throughout the study.

### 2.4 Treatment Administration

Nine different treatments were implemented in this study. Firocoxib (57 mg tablets; Equioxx Tablets; Boehringer Ingelheim Animal Health, Duluth, GA), meloxicam (15 mg tablets; Zydus Pharmaceuticals Inc., Pennington, NJ), and flunixin transdermal (50 mg/mL; Banamine Transdermal, Merck Animal Health, Madison, NJ) were each given at three different dosages. Each drug‐dosage combination was considered a treatment, totaling nine treatments.

Goats in this trial were simultaneously enrolled in a concurrent study investigating the analgesic effects of each treatment on goats with experimentally induced lameness. Each goat received an intra‐articular joint injection into the left hind limb and right hind limb with a dose of amphotericin B (5 mg/0.25 mL). A 14‐day washout period (14d) occurred between lameness induced in the left hind limb and the right hind limb. This lameness induction model was chosen based on previously published literature (Weeder et al. 2025). Each goat received two treatments during this crossover design trial. One treatment was given in the first phase, a washout period occurred, and then each goat was given a second treatment in phase two. At least 14 days were allowed for a washout between treatments. PO treatments were administered in a gelatin capsule using a balling gun, while TD treatments were applied with a syringe directly on the skin. PO of firocoxib and meloxicam treatments were rounded to the nearest half tablet based on dosage calculations. The treatments used were:

- **FIRO‐LOW (*n* = 8)**: Firocoxib tablet at 0.5 mg/kg.
- **FIRO‐MID (*n* = 8)**: Firocoxib tablet at 1.0 mg/kg.
- **FIRO‐HIGH (*n* = 8)**: Firocoxib tablet at 2.0 mg/kg.
- **MEL‐LOW (*n* = 8)**: Meloxicam tablet at 1.0 mg/kg.
- **MEL‐MID (*n* = 8)**: Meloxicam tablet at 2.0 mg/kg.
- **MEL‐HIGH (*n* = 8)**: Meloxicam tablet at 3.0 mg/kg.
- **FLU‐LOW (*n* = 8)**: Flunixin transdermal at 3.3 mg/kg.
- **FLU‐MID (*n* = 8)**: Flunixin transdermal at 4.2 mg/kg.
- **FLU‐HIGH (*n* = 8)**: Flunixin transdermal at 5.0 mg/kg.

Oral formulations of firocoxib and meloxicam were chosen over parenteral routes due to ease of administration and practicality from a producer standpoint. Low dosages for each drug were chosen based on previous research or approvals in adult cattle, calves, and sheep (US FDA 2017; Stock et al. 2013, 2014). Mid and high‐range dosages were chosen with the knowledge that goats metabolize drugs at a higher rate than calves, cattle and sheep, which insinuated higher drug dosages for investigation (Myers et al. 2021). Dosages were chosen based on current knowledge and experience of a board‐certified veterinary pharmacologist (MDK). Control goats (*n* = 8) were utilized for the concurrent lameness trial that occurred during this study, but were not utilized for pharmacokinetic analysis. This contributed to the overall animal numbers (Weeder et al. 2025).

### 2.5 Plasma Sample Collection

Intravenous jugular catheters (16G × 7.5 cm, Mila International Inc., Florence, KY, US) were placed aseptically by a licensed veterinarian 12 h prior to the start of the study in either the left or right jugular vein. Catheters were flushed prior to and after sample collection using heparinized saline at a rate of 10iu per 1 mL (Medefil, Glendale Heights, IL, US) to maintain catheter patency. After the 24 h timepoint, catheters were removed, and samples were collected via jugular venipuncture (alternating left and right sides for animal comfort). Blood samples were collected at the following timepoints: −24, 0, 1, 2, 3, 4, 5, 6, 8, 10, 12, 24, 36, 48, 60, and 72 h. Approximately 3 mL of blood was collected at each timepoint and placed into lithium heparin vacutainer tubes (BD Medical, Franklin Lakes, NJ, USA). Blood samples were centrifuged at 1500 g for 15 min after collection. Separated plasma was pipetted into 2 mL cryovials (VWR International, Radnor PA, USA) and stored at −80°C.

### 2.6 Determination of Plasma Drug Concentrations: UHPLC–MS/MS

#### 2.6.1 Chemicals and Materials

Analytical standards of meloxicam, meloxicam‐d3, flunixin, flunixin‐d3, and firocoxib were purchased from the Cayman Chemical Company (Ann Arbor, MA), while firocoxib‐d6 was purchased from Alfa Chemistry (Holbrook, NY). Optima LC–MS grade acetonitrile, formic acid, methanol, water, and HPLC grade phosphoric acid were provided by Fisher Chemical (Ottawa, Ontario, CA). The LC–MS grade dimethyl sulfoxide was supplied by Thermo Scientific (Waltham, MA). Untreated, blank goat plasma in lithium heparin was obtained from Lampire Biological Laboratories (Pipersville, PA). Oasis PRiME HLB 96 well μElution plates were purchased from Waters Corporation (Milford, MA).

#### 2.6.2 UHPLC–MS/MS Sample Preparation and Extraction

Plasma samples were thawed at room temperature (23°C) prior to analysis start. A mixed analyte stock standard solution containing firocoxib (FIRO), meloxicam (MEL), and flunixin (FTD) was prepared at 3.00E+02, 10.0, and 10.0 μg/mL, respectively, in dimethyl sulfoxide (DMSO) and stored at −20°C. A mixed internal standard (IS) stock solution containing firocoxib‐d6 (FIRO‐d6), meloxicam‐d3 (MEL‐d3), and flunixin‐d3 (FTD‐d3) was prepared at 40.0, 10.0, and 10.0 μg/mL, respectively, in DMSO and stored at −20°C.

The total volumes of the prepared study, daily mixed calibration, and daily mixed quality control (QC) samples were 300.0 μL each. All samples consisted of goat plasma, mixed IS solution, and either aqueous phosphoric acid (4%, v/v) diluent—for study samples—or mixed analyte standard in aqueous phosphoric acid (4%, v/v)—for daily mixed calibration and QC samples. Specifically, the daily mixed calibration and QC samples included 100.0 μL of blank goat plasma, while the study samples included the goat plasma of interest at identical volumes. All samples were spiked with mixed IS solution and diluted appropriately with phosphoric acid (4%, v/v aq) to contain 3.00E+02, 75.0, and 75.0 ng/mL of FIRO‐d6, MEL‐d3, and FTD‐d3, respectively. The daily mixed calibration samples produced calibration curves for FIRO, MEL, and FTD by appropriate phosphoric acid (4%, v/v aq) dilutions to the mixed analyte stock. The FIRO calibration curves were prepared at the following concentrations: 0.750, 1.50, 3.00, 7.50, 15.0, 30.0, 75.0, 150, 300, 750, 1500, 2250, and 3000 ng/mL. The MEL calibration curves were prepared at the following concentrations: 0.0250, 0.0500, 0.100, 0.250, 0.500, 1.00, 2.50, 5.00, 10.0, 25.0, 50.0, 75.0, and 100 ng/mL. The FTD calibration curves were prepared at the following concentrations: 0.0250, 0.0500, 0.100, 0.250, 0.500, 1.00, 2.50, 5.00, 10.0, 25.0, 50.0, 75.0, and 100 ng/mL. The standard concentrations for the daily mixed calibration samples were established to three significant figures. The daily mixed QC samples contained FIRO, MEL, and FTD mixed analyte stock diluted appropriately by phosphoric acid (4%, v/v aq). The QC concentrations of FIRO were 1.50, 30.0, 300, and 2250 ng/mL. The QC concentrations of MEL were 0.0500, 1.00, 10.0, and 75.0 ng/mL. The QC concentrations of FTD were 0.0500, 1.00, 10.0, and 75.0 ng/mL. The standard concentrations for the daily mixed QC samples were established to three significant figures. Study samples that exceeded the method's linear calibration range were diluted as needed with 4% (v/v) aqueous phosphoric acid before extraction and reanalysis. All samples were centrifuged at 1300 × g for 20 min at 20°C using a Thermo Scientific Sorvall ST 16R centrifuge.

Using a positive pressure manifold, the supernatants were subjected to reversed‐phase solid‐phase extraction (RP‐SPE) with Oasis PRiME HLB μElution plates (Corporation, W 2024) according to the protocol in Table S1.

#### 2.6.3 UHPLC–MS/MS Instrumentation

The simultaneous quantitation of FIRO, MEL, and FTD in caprine plasma was performed on a Waters Acquity Ultra Performance Liquid Chromatography H‐Class PLUS‐tandem Xevo TQ‐S Mass Spectrometry instrument. The chromatographic conditions and gradient elution program are described in Tables S2 and S3, respectively. The mass spectrometric source conditions and multiple reaction monitoring (MRM) detection parameters are reported in Tables S4 and S5, respectively. Quantitative data were acquired and processed using TargetLynx operating software.

#### 2.6.4 Method Validation

The developed UHPLC–MS/MS method to simultaneously quantitate FIRO, MEL, and FTD in caprine plasma adhered to the FDA's *Bioanalytical Method Validation Guidance for* Industry report (FDA, U.S. 2018). The calibration curves, with logarithmic axis transformation, ranged from [1.50E+00, 5.00E‐02, 5.00E‐02] to [3.00E+03, 1.00E+02, 1.00E+02] in ng/mL for FIRO, MEL, FTD, respectively, and exhibited coefficients of determination (R^2^) consistently greater than 0.990. Due to daily mixed QC accuracy and reproducibility, the limits of quantitation (LOQ) were 1.50, 1.00, and 1.00 ng/mL for FIRO, MEL, and FTD, respectively. The method was validated by an analysis of the daily mixed QC samples; the average percent recovery, average intra‐day precision—percent relative standard deviation (% RSD)—and inter‐day precision (% RSD) results are in Table S5.

### 2.7 Pharmacokinetic Analysis

Pharmacokinetic analysis for each goat was performed using computer software (PK Solver). For each goat, the plasma drug concentrations over time were analyzed using noncompartmental analysis with uniform weighting based on the statistical moment theory (Gibaldi and Perrier 1982). The individual animal pharmacokinetics were determined for each drug (firocoxib, meloxicam or flunixin transdermal) and dose level (Low, Mid, High). The terminal rate constant (λ_z_) was determined by linear regression using the last 3–5 time points of the log plasma concentration‐time curve. The terminal rate constant (λ_z_) was used to calculate the terminal half‐life (T_½_). The area under the plasma concentration drug curve (AUC) was calculated using the trapezoidal method. To determine AUC_0‐∞_, the last measurable drug concentration was extrapolated to infinity. The time to maximum concentrations (T_max_) and maximum concentrations (C_max_) were determined by visual inspection of the data. Descriptive statistics are reported.

## 3 Results

No adverse physiological or behavioral effects were noted in animals enrolled in the trial given firocoxib, meloxicam, or transdermal flunixin at the tested dosages.

**FIGURE 1:**
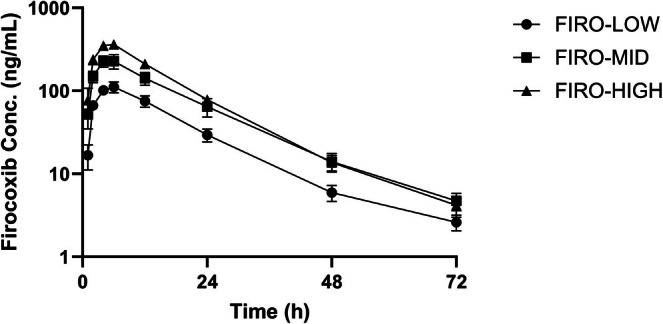
Plasma concentrations of firocoxib in goats following oral administration at three dosages. Goats were administered oral firocoxib at 0.5 mg/kg (FIRO‐LOW), 1.0 mg/kg (FIRO‐MID), or 2.0 mg/kg (FIRO‐HIGH) after induction of amphotericin B–induced lameness. Plasma drug concentrations were measured over time to assess pharmacokinetic profiles and systemic exposure.

**FIGURE 2:**
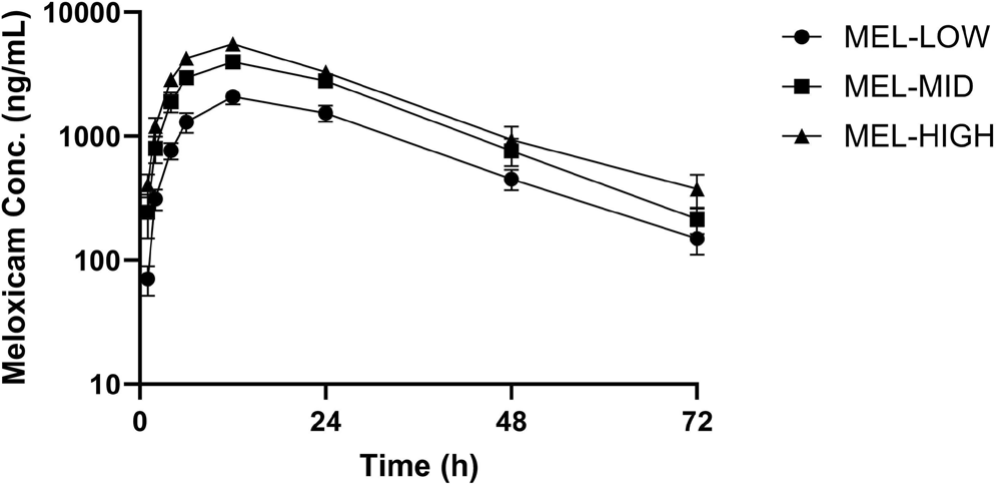
Plasma concentrations of meloxicam in goats following oral administration at three dosages. Goats were administered oral meloxicam at 1.0 mg/kg (MEL‐LOW), 2.0 mg/kg (MEL‐MID), or 3.0 mg/kg (MEL‐HIGH) after induction of amphotericin B–induced lameness. Plasma drug concentrations were measured over time to evaluate absorption and systemic exposure.

**FIGURE 3:**
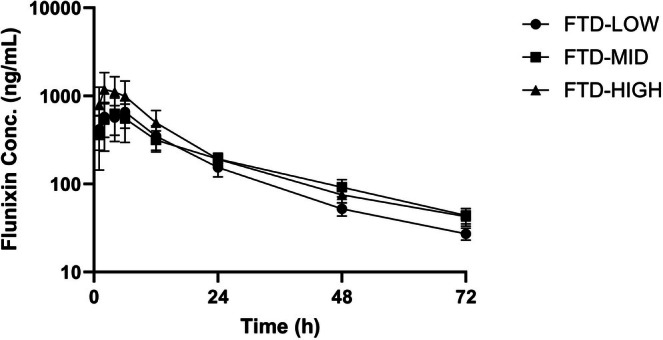
Plasma concentrations of transdermal flunixin in goats following application at three dosages. Goats received transdermal flunixin at 3.3 mg/kg (FTD‐LOW), 4.2 mg/kg (FTD‐MID), or 5.0 mg/kg (FTD‐HIGH) after induction of amphotericin B–induced lameness. Plasma drug concentrations were measured over time to determine pharmacokinetic behavior and potential analgesic exposure.

Pharmacokinetic parameters for each of the three dosages of PO firocoxib, PO meloxicam, and TD flunixin following single administration in meat‐type castrated goats are summarized as mean values with corresponding ranges in Table 1. Mean plasma concentrations over time for firocoxib dosages are shown in Figure 1, for meloxicam dosages in Figure 2, and for flunixin transdermal dosages in Figure 3.

**TABLE 1:** Pharmacokinetic outcome parameters of firocoxib, meloxicam, and flunixin for goats with amphotericin‐B induced lameness and then analgesic treatment.

Parameter^a^	Unit	FIRO		
		0.5 mg/kg (*n* = 8)	1.0 mg/kg (*n* = 8)	2.0 mg/kg (*n* = 8)
T_1/2_	h	9.1	10.2	9.2
		6.9–13.4	6.4–14.7	6.8–12.8
T_max_	h	5.4	4.7	4.9
		4–6	4–6	4–6
C_max_	ng/mL	106.7	200.7	381.5
		65.8–205.4	140.2–531.8	331.2–431.7
AUC_0–∞_	ng/mL × h	1948.1	4102.7	6237.8
		898–3790	2747–10,458	5018–7954
AUC Extrapolated	%	0.97	0.98	0.99
		0.90–0.99	0.96–0.99	0.98–1.00
MRT_0–∞_	h	14.9	16.0	13.9
		11.6–20.7	11.2–21.6	9.9–19.0

Parameter^a^	Unit	MEL		
		1.0 mg/kg (*n* = 8)	2.0 mg/kg (*n* = 8)	3.0 mg/kg (*n* = 8)
T_1/2_	h	13.3	12.2	11.7
		10.1–22.3	9.8–15.4	8.3–20.5
T_max_	h	13.3	13.1	11.0
		12–24	12–24	6–12
C_max_	ng/mL	2040.3	3891.7	5511.7
		1431.4–3709.1	2762.5–5240.9	3707.5–7611.5
AUC_0–∞_	ng/mL × h	66,547	122,035	160,916
		43,029–108,659	79,737–211,993	99,684–262,293
AUC Extrapolated	%	0.95	0.97	0.96
		0.89–0.99	0.94–0.99	0.89–1.00
MRT_0–∞_	h	26.3	23.8	22.6
		19.9–38.5	20.4–30.4	14.7–35.4

Parameter^a^	Unit	FTD		
		3.3 mg/kg (*n* = 8)	4.2 mg/kg (*n* = 8)	5.0 mg/kg (*n* = 8)
T_1/2_	h	16.5	22.0	17.8
		10.7–62.0	16.6–67.4	7.4–56.3
T_max_	h	6.4	10.5	5.1
		2–24	4–24	2–24
C_max_	ng/mL	407.6	436.7	600.7
		63.9–2085.7	103.9–1654.8	108.1–5602.4
AUC_0–∞_	ng/mL × h	11,699.2	14,813.7	18,182.4
		6759–32,184	9218–24,691	11,244–61,127
AUC Extrapolated	%	0.84	0.83	0.81
		0.51–0.98	0.51–0.98	0.57–0.99
MRT_0–∞_	h	26.4	32.0	29.0
		12.0–97.1	11.3–100.8	8.9–85.9

*Note:* Harmonic means are presented for T_1/2_. All other means are geometric means. Treatments modeled included oral (PO) firocoxib at 0.5 mg/kg (*n* = 8), PO firocoxib at 1.0 mg/kg (*n* = 8), PO firocoxib at 2.0 mg/kg (*n* = 8), PO meloxicam at 1.0 mg/kg (*n* = 8), PO meloxicam at 2.0 mg/kg (*n* = 8), PO meloxicam at 3.0 mg/kg (*n* = 8), transdermal (TD) flunixin at 3.3 mg/kg (*n* = 8), TD flunixin at 4.2 mg/kg (*n* = 8), and TD flunixin at 5.0 mg/kg (*n* = 8).

^a^ T_1/2_ is elimination half‐life, T_max_ is time to Cmax, C_max_ is maximum plasma concentration, AUC_0–∞_ is percent of the AUC extrapolated, AUC is extrapolated area under the curve extrapolated to infinity, MRT_0–∞_ is mean residence time extrapolated to infinity.

Method validation results for LC–MS analysis for each drug are summarized in Table 2.

**TABLE 2:** Method validation results based on daily mixed QC sample analysis.

Analyte	Nominal concentration (ng/mL)	Average recovery (%), *n* = 3	Average intra‐day precision (% RSD), *n* = 3	Inter‐day precision (% RSD), *n* = 5
MEL	1.00E+00	9.22E+01	1.98E+00	9.00E+00
	1.00E+01	9.83E+01	1.90E+00	3.47E+00
	7.50E+01	9.87E+01	1.47E+00	2.06E+00
FTD	1.00E+00	9.50E+01	2.34E+00	6.57E+00
	1.00E+01	9.71E+01	2.44E+00	3.37E+00
	7.50E+01	1.00E+02	1.73E+00	2.45E+00
FIRO	1.50E+00	9.74E+01	3.71E+00	9.38E+00
	3.00E+01	1.01E+02	5.29E+00	8.27E+00
	3.00E+02	1.00E+02	2.03E+00	3.23E+00
	2.25E+03	9.80E+01	3.24E+00	4.72E+00

*Note:* Analytes evaluated were meloxicam (MEL), flunixin (FTD), and firocoxib (FIRO).

## 4 Discussion

This is the first study to describe the pharmacokinetics of PO firocoxib, PO meloxicam, and TD flunixin at varying dosages in healthy, adult, castrated male crossbred goats with experimentally induced lameness. No adverse effects were noted in any animals enrolled following the administration of test articles.

The lack of any FDA‐approved drug for pain mitigation in goats inhibits veterinarians and producers' abilities to provide proper analgesia for caprine patients. The only approved analgesic for any livestock species in the United States is TD flunixin at 3.3 mg/kg in cattle (US FDA 2017), which was chosen as the low dose in this study. Firocoxib is currently approved in the United States for use in dogs and horses (McCann et al. 2004) and meloxicam is approved for use in humans and small animals (de la Puente et al. 2024; Ingelheim 2025). All previously mentioned drugs are easily accessible to producers through veterinarian prescription, which elicited their selection for comparison in this study. Compared to other livestock species, goats have shown to metabolize drugs at a faster rate (Myers et al. 2021), which prompted the evaluation of varying dosages for all drugs used in this study.

The pharmacokinetics of firocoxib have been previously described in goats. In a study by Stuart et al. (2019), firocoxib administered orally at a 0.5 mg/kg resulted in a T_1/2_ of 21.51 h (range: 10.21–48.32 h) and a C_max_ of 139 ng/mL (range: 87–196 ng/mL). In contrast, our study observed a lower T_1/2_ and C_max_ at the same dosage and administration route, which may indicate more rapid elimination and reduced peak exposure. Possible explanations for variation between studies may include female vs. male sex differences, breed differences (mixed breed vs. meat type goats), and age differences (2.5 years vs. 9–12 m). Goats in this study also underwent an experimentally induced lameness model, which also must be taken into consideration when comparing pharmacokinetic parameters to other studies. To the authors' knowledge, no other studies have reported the pharmacokinetics of firocoxib in goats at doses of 1.0 mg/kg or 2.0 mg/kg. Additionally, there are no published data regarding IC50, IC80, or prostaglandin E/metabolite values after firocoxib administration in goats, limiting pharmacodynamic comparisons.

Meloxicam pharmacokinetics have been previously reported at lower dosages and different routes of administration in goats when compared to our study. Studies investigating a meloxicam dose of 0.5 mg/kg PO produced T_1/2_ values of 10.7 h (range: 7–14.6 h) (Bublitz et al. 2019), 10.69 ± 1.49 h (Karademir et al. 2016) and 11.8 ± 1.7 h (Ingvast‐Larsson et al. 2011). These T_1/2_ values were similar to those found in the present study at a 3.0 mg/kg dose, suggesting consistent elimination patterns across dosages. To date, meloxicam at doses of 1.0, 2.0, or 3.0 mg/kg in goats has not yet been reported. Additionally, no caprine IC50, IC80, or prostaglandin inhibition data are currently available.

Pharmacokinetics of transdermal (TD) flunixin in goats, administered at the US cattle‐approved dose of 3.3 mg/kg, has been investigated across several studies. Reported elimination half‐lives (T_1/2_) vary widely: Reppert et al. (2019) documented the longest mean T_1/2_ at 43.12 h (range: 15.98–62.49 h), while Graves et al. (2020) reported a much shorter value of 7.65 ± 2.14 h, and Meira et al. (2022) observed an intermediate value of 21.63 h (range: 14.99–30.32 h). When comparing reported results to parameters from our study at 3.3 mg/kg, our data were most similar to those reported in Graves et al. (2020). As of publication, there are no reported pharmacokinetic parameters of TD flunixin at 4.2 or 5.0 mg/kg in goats. Pharmacokinetic results among all dosages of TD flunixin in this study showed considerable variability. Varying results from this study and previous studies regarding pharmacokinetic parameters of TD flunixin may be due to differences regarding inadequate adsorption (oral ingestion from licking, drug distribution to environment, etc.), poor drug administration technique, differences in skin and hair coat characteristics, body fat percentages, or unknown factors. When considering these differences, the authors also acknowledge that goats in this study underwent experimentally induced lameness. The effect of this lameness model on pharmacokinetic parameters is unknown when comparing results to other reported pharmacokinetic parameters in goats. In Reppert et al. (2019), prostaglandin E₂ (PGE₂) concentrations decreased by 100% following intravenous (IV) flunixin administration, but only by 50% following TD administration. The mean IC₈₀ for PGE₂ inhibition was 0.28 μg/mL (range: 0.08–0.69 μg/mL) (Reppert et al. 2019), which may indicate a concentration‐dependent anti‐inflammatory effect.

Results from this study suggest that dose linearity across all drugs used is maintained from the low dose to the intermediate dose but not going from the intermediate dose to the high dose. Specifically, firocoxib at a dose of 2.0 mg/kg and meloxicam at a dose of 3.0 mg/kg did not reach expected concentrations; linearity was assumed. One possible explanation is that firocoxib and meloxicam absorption in the gastrointestinal tract at higher dosages is overwhelming, leading to drug loss in feces excretion.

When examining pharmacokinetic parameters from this study and results from the concurrent lameness study assessment in these goats (Weeder et al. 2025), recommendations include flunixin transdermal at a dose of 3.3 or 5.0 mg/kg, or meloxicam at a dose of 2.0 mg/kg. All three flunixin transdermal dosages used in this study maintained a longer T_1/2_ and MRT_0‐∞_ compared to all meloxicam and firocoxib dosages. When analyzing the graphical representation of flunixin transdermal plasma concentrations with the reported IC80 of 0.28 μg/mL, data show that plasma concentrations fall below 0.28 μg/mL between the 12 and 24 h datapoints. This suggests that flunixin transdermal at either concentration would need to be administered twice daily in goats. FDA approval of flunixin transdermal in cattle for foot rot pain also makes this drug an attractive option for veterinarians to use in other livestock species like goats. Recommendations based on previous work in pain evaluation and analgesic intervention in experimentally induced lameness in goats also support the use of flunixin transdermal for analgesic intervention in caprine patients (Weeder et al. 2025).

There is no current IC_80_ data for meloxicam in goats or in cattle. Without this information, relevant dosing recommendations cannot be made for any of the dosages investigated in this study. As this goal was not an immediate concern of this study, more prostaglandin inhibition data are necessary before accurate dosing intervals can be made for any of the three meloxicam dosages investigated.

The UHPLC–MS/MS method used in this study is novel because of its ability to simultaneously quantify FIRO, MEL, and FTD in caprine plasma. The LOQ was below the physiologically relevant levels of the analytes; thus the method was sufficient for the study requirements. (Shukla et al. 2007; Stuart et al. 2019; Königsson et al. 2003; Young et al. 2022; Fogle et al. 2020) In the past, FIRO, MEL, and FTD levels in caprine plasma have been individually analyzed using single‐analyte methods (Shukla et al. 2007, Stuart et al. 2019, Königsson et al. 2003).

Analytical methods have been reported to simultaneously and individually measure FIRO, MEL, and FTD in equine plasma (Young et al. 2022; Fogle et al. 2020). In comparison, this study's method offers the following benefits: low plasma volume demands, short analysis time, and high throughput potential. Specifically, this study method calls for 100 μL of plasma; this sample preservation ensures analysis is accomplished without excessive sample procurement from the animal and allows for additional analyses to be performed. This method can produce analytical results in around 10 min per sample from sample prep to analyte quantification because of its straightforward sample preparation and solid phase extraction protocol. The present method was developed with a 96‐sample well format, which allows for batch processing with automated sample injection and miniaturized sample preparation. Furthermore, the high throughput method was developed with conventional LCMS parameters which opens up the potential for additional analytes to be included in the simultaneous method if desired.

Overall, flunixin transdermal at a dose of 3.3 or 5.0 mg/kg is recommended to treat pain in goats presenting for lameness based on pharmacokinetic and pain‐related research. Results suggest further pharmacokinetic research is needed to determine the optimal dosing strategies for firocoxib, meloxicam, and transdermal flunixin in goats. Future studies should include pharmacokinetic research in both meat and dairy type breeds, implement goats of varying ages, goats of both sexes, and goats not enrolled in an experimentally induced lameness trial. Determining the pharmacodynamics via inhibition of COX enzyme products, such as prostaglandin, would further enhance the understanding of these drugs and the doses used.

Although additional research is needed, data from this study help decrease the current paucity of literature surrounding the pharmacokinetics of commonly used veterinary NSAIDs in goats. Strengths of this study include implementation of three commonly used NSAIDs at three varying dosages, and development of a novel LCMS method to evaluate goat plasma for firocoxib, meloxicam, and flunixin simultaneously. Limitations include lack of IV comparisons for each drug and dosage combination for determination of bioavailability, exclusion of goats of different age, breed, or sexes, and restriction of only PO and TD administration routes.

## Author Contributions

M.D.K., E.J.R., P.D., and J.F.C. contributed to study design. M.M.W., R.T., M.B., A.K.C., A.A.N., and B.R.F. performed the experiments. C.T.C., K.K., R.T., M.B., and M.M.W. performed data analysis. M.M.W. composed the manuscript with help from M.D.K., E.J.R., and R.T. All authors have read and approved the final document.

## Funding

This work was supported by the U.S. Department of Agriculture, 2020‐67015‐31546.

## Ethics Statement

The authors confirm that the ethical policies of the journal, as noted on the journal's author guidelines page, have been adhered to. The authors confirm that they have adhered to the US standards for the protection of animals used for scientific research.

## Conflicts of Interest

The authors declare no conflicts of interest.

## Supporting information


**Appendix S1:** jvp70035‐sup‐0001‐AppendixS1.docx.

## Data Availability

The data that support the findings of this study are available from the corresponding author upon reasonable request.
