# Supplementary material for: Pharmacokinetic Parameters of Oral Firocoxib, Oral Meloxicam, and Transdermal Flunixin in Meat Type Goats
Source: J Vet Pharmacol Ther. 2025 Dec 1;49(2):172–9. doi: 10.1111/jvp.70035 (PMC12968514; doi:10.1111/jvp.70035)
Supplement: Supplementary file 1 — Appendix S1: jvp70035‐sup‐0001‐AppendixS1.docx. [file JVP-49-172-s001.docx]

**Supplemental Table 1.** Solid phase extraction protocol facilitated by Oasis hydrophilic-lipophilic sorbent and positive pressure assistance.

| Step # | Strategy type | Strategy details | Volume (µL) |
| --- | --- | --- | --- |
| 1 | Sample introduction | Pre-treated sample | 300.0 |
| 2 | Hydrophilic wash | Water: methanol (90:10, v/v) | 300.0 |
| 3 | Organic elution | Acetonitrile: methanol (90:10, v/v) | 50.00 |
| 4 | Direct eluate dilution | Formic acid in water (0.1%, v/v) | 50.00 |

**Supplemental Table 2**. Chromatographic conditions for the Waters Acquity Ultra Performance Liquid Chromatography H-Class PLUS system.

| Autosampler temperature (°C) | 5.0 |
| --- | --- |
| Injection volume (µL) | 2.00 |
| Column | Acquity UPLC C18 HSS T3 (100 Å, 1.8 µm, 2.1 x 50 mm) |
| Pre-column | Acquity UPLC C18 HSS T3 VanGuard (100 Å, 1.8 µm, 2.1 x 5 mm) |
| Column temperature (°C) | 40.0 |
| Mobile phase | A: Formic acid in water (0.1%, v/v) |
|  | B: Formic acid in acetonitrile (0.1%, v/v) |

**Supplemental Table 3**. Gradient elution program with the mobile phase composed of 0.1%, v/v formic acid in water (A) and 0.1%, v/v formic acid in acetonitrile (B).

| Time (min.) | Mobile phase (%) | | Flow rate (mL/min.) |
| --- | --- | --- | --- |
|  | A | B |  |
| 0.00 | 95.0 | 5.00 | 0.400 |
| 4.00 | 5.00 | 95.0 |  |
| 5.00 | 5.00 | 95.0 |  |
| 5.01 | 95.0 | 5.00 |  |
| 7.00 | 95.0 | 5.00 |  |

**Supplemental Table 4**. Mass spectrometric source conditions for the Waters Xevo TQ-S tandem Mass Spectrometry system.

| Capillary voltage (kV) | 3.30 |
| --- | --- |
| Source temperature (°C) | 150 |
| Desolvation -nitrogen- gas flow (L/hr.) | 800 |
| Desolvation temperature (°C) | 200 |
| Cone -nitrogen- gas flow (L/hr.) | 150 |
| Collision -argon- gas flow (mL/min.) | 0.15 |

**Supplemental Table 5**. Targeted MRM detection parameters for collision-induced fragmentation. Analytes were meloxicam (MEL), meloxicam-d3 (MEL-d3), flunixin (FTD), flunixin-d3 (FTD-d3), firocoxib (FIRO), and firocoxib-d6 (FIRO-d6).

| Compound | Precursor ion (m/z) | Product ion (m/z) | Transition classification | Cone energy (V) | Collision energy (V) | Electrospray ionization mode |
| --- | --- | --- | --- | --- | --- | --- |
| MEL | 351.9851 | 115.0434 | Quantifier | 48 | 20 | Positive |
|  |  | 140.9734 | Qualifier |  | 18 |  |
| MEL-d3 | 354.9851 | 140.9764 | Quantifier | 44 | 20 |  |
|  |  | 187.0535 | Qualifier |  | 14 |  |
| FTD | 297.0166 | 264.0135 | Quantifier | 6 | 32 |  |
|  |  | 279.0269 | Qualifier |  | 22 |  |
| FTD-d3 | 300.0127 | 112.1246 | Qualifier | 30 | 46 |  |
|  |  | 264.0180 | Quantifier |  | 32 |  |
| FIRO | 337.0489 | 129.9968 | Qualifier | 24 | 30 |  |
|  |  | 283.0431 | Quantifier |  | 8 |  |
| FIRO-d6 | 343.0889 | 135.8492 | Qualifier | 26 | 28 |  |
|  |  | 289.0823 | Quantifier |  | 8 |  |
